# Usage of cell nomenclature in biomedical literature

**DOI:** 10.1186/s12859-017-1978-0

**Published:** 2017-12-21

**Authors:** Şenay Kafkas, Sirarat Sarntivijai, Robert Hoehndorf

**Affiliations:** 1 Computational Bioscience Research Center, Computer, Electrical and Mathematical Sciences & Engineering Division, King Abdullah University Science and Technology, 4700 KAUST, Thuwal, 23955-6900 Saudi Arabia; 2 The European Bioinformatics Institute (EMBL-EBI), European Molecular Biology Laboratory, Wellcome Genome Campus, Hinxton, Cambridge, SD CB10 1 UK

**Keywords:** Cell nomenclature, Text mining, Cell lines, Cell types, Ontologies

## Abstract

**Background:**

Cell lines and cell types are extensively studied in biomedical research yielding to a significant amount of publications each year. Identifying cell lines and cell types precisely in publications is crucial for science reproducibility and knowledge integration. There are efforts for standardisation of the cell nomenclature based on ontology development to support FAIR principles of the cell knowledge. However, it is important to analyse the usage of cell nomenclature in publications at a large scale for understanding the level of uptake of cell nomenclature in literature by scientists. In this study, we analyse the usage of cell nomenclature, both in Vivo, and in Vitro in biomedical literature by using text mining methods and present our results.

**Results:**

We identified 59% of the cell type classes in the Cell Ontology and 13% of the cell line classes in the Cell Line Ontology in the literature. Our analysis showed that cell line nomenclature is much more ambiguous compared to the cell type nomenclature. However, trends indicate that standardised nomenclature for cell lines and cell types are being increasingly used in publications by the scientists.

**Conclusions:**

Our findings provide an insight to understand how experimental cells are described in publications and may allow for an improved standardisation of cell type and cell line nomenclature as well as can be utilised to develop efficient text mining applications on cell types and cell lines. All data generated in this study is available at https://github.com/shenay/CellNomenclatureStudy.

## Background

Distinct identification of cell lines and cell types in the literature is important for the reproducibility of studies in order to clearly identify and differentiate information about the cell types or cell lines used in an experiment. The Cell Ontology (CL) [1] and the Cell Line Ontology (CLO) [2] are ontologies that have been developed to provide a formal representation of cell types and cell lines, and therefore provide a resource that naturally enables standardization of nomenclature. Both ontologies are developed as part of the Open Biomedical Ontologies (OBO) [3] initiative and interoperate with the growing set of ontologies developed within the OBO. The Human Cell Atlas (HCA) [4] is a complementary effort to the development of ontologies and aims to cover the characteristics, including function and anatomical location, of all human (and to a lesser degree mammalian) cells. The HCA effort will result in high-dimensional compendium of information about cells that are found both in vitro and in vivo. To capture and describe such detailed information about cells will require a comprehensive metadata model for which already existing ontologies provide a natural solution. In particular, ontologies such as CL and CLO are already widely applied for annotation of different datasets, and reuse of these ontologies has the potential to significantly extend the utility of any new dataset that reuses these ontologies, since data can be combined semantically. It is crucial to understand the landscape of the usages of cell types and cell lines nomenclature by biomedical researchers in order to establish a robust framework to expand CL and CLO and allow both ontologies to cover a wide range of phenomena.

Here, we perform a large-scale analysis focusing on cell nomenclature usage in the biomedical literature by using text mining methods. Our aims are to understand how the nomenclature related to cell types and cell lines is used in literature, how both evolved over time, and how this information may allow us to provide better tools and resources for biomedical researchers. To the best of our knowledge, this is the first study with this focus, while several previous studies focused on identifying the cell type and cell line names in text [5, 6].

## Methods

### Resources used

Latest archived version of the Open Access (OA) full text articles (http://europepmc.org/ftp/archive/v.2017.06/) (~1.5 million) from Europe PubMed Central [7] is used as the literature collection in the analyses.

We compiled two dictionaries for in vivo cells from the CL and in vitro cells from the CLO to annotate cell type and cell line text tokens in publications respectively. The cell type dictionary is generated from the labels and synonyms used in the CL, obtained from the CL’s OWL version available at http://purl.obofoundry.org/obo/cl.owl. The cell line dictionary is generated from the CLO’s OWL version available at http://purl.obofoundry.org/obo/clo.owl. Both ontologies were downloaded on 19 May 2017. There exist no widely accepted rules on how to name cell lines, thus resulting in a non-standardised vocabulary of cell lines as described in [8]. A number of ambiguous cell line names are still in use until today. Therefore, aiming for precision over recall, we applied a refinement process to the dictionaries before using them to reduce false positive calls. In this process, we semi-automatically filtered out the terms that would introduce potentially high numbers of false positives. These are the terms containing less than 3 characters for cell types and less than 4 characters for cell lines.

We further remove digits as well as the generic terms (e.g., P1, mouse cell, cell line, human, mammalian). We removed a total number of 9 cell type terms and 650 cell type terms from the dictionaries. The final cell type dictionary consists of 3838 term referring to 2180 distinct cell types while the cell line dictionary consists of 76,747 terms belonging to 38,605 distinct cell lines.

### Annotating cell lines and cell types in text

We used the Whatizit [9] entity recognition pipeline to annotate cell type and cell line names in the Open Access full text articles by using our dictionaries on cell types and cell lines. Whatizit employs taggers based on finite automata and the MAchine Learning for LanguagE Toolkit (MALLET) [10]. The taggers of Whatizit annotate documents in a dictionary-based approach.

## Results and discussion

### Manual analysis

We conducted manual analyses on the annotated corpora to evaluate the performance of our text mining approach as well as to understand how the quality of terminological resources on cells like CL and CLO might be improved. To this end, we generated two evaluation sets, each of them comprising randomly selected 50 sentences containing either at least one annotation or a specific keyword, “cell type” and “cell line” for CL and CLO annotation evaluation respectively.

Annotation errors were due to false positive and false negative cell type/line name callings. Identifying cell lines is a non-trivial problem due to the following challenges:

- Many cell line names consist of only numbers. (e.g. 2–2, 548)
- Many cell line names consist of less than 4 letters (e.g. C2, S2)
- Cell line names often look like gene/protein names (e.g. MCF2)
- Some cell line names look like person names (e.g. Ishikawa)
- Some cell line names are very generic (e.g. adapted, focus, label)

Although we removed several potentially ambiguous terms from the cell line dictionary in the refinement process (see Section 2.1) as they might introduce a high number of false positives into our analyses, we still have false positive annotations of cell lines. For example, ‘ARL6’ is both a cell line name and a gene name leading to a false positive annotation in some of the articles.

Missing cell line names (false negatives) are mainly due to the cell lines which are in CLO’s scope but not yet been incorporated in a CLO release. For example, NCI-60 or LINCS cell lines are currently missing in CLO and cannot be found using our dictionary-based approach. Other missing cell lines are due to their uncovered synonyms by CLO. For example, the cell line name ‘HEK293T’ is not found in literature since the cell line is referred to as ‘293 T’ in CLO and the ontology does not contain the synonym ‘HEK293T’. An additional check revealed that, ‘HEK293T’ exists in the Cellosaurus [11]. Our cell line tagger achieved a precision value of 82.22%, a recall value of 72.55% and an F-score value of 77.08%. Results indicate that the high ambiguity in the cell line naming plays a role in identifying cell lines in text precisely. Furthermore, there is still some room for expanding the CLO’s coverage, both in terms of additional labels or synonyms as well as in terms of missing classes, by incorporating additional resources on cell lines such as the Cellosaurus.

Cell type nomenclature used for the cell type names is much less ambiguous compared to the cell line nomenclature. Nevertheless, some false positives occur due to overlapping cell type and anatomy terms such as ‘smooth muscle’. Other false positives are due to abbreviations. For example, SMC is used as an abbreviation for ‘Smooth muscle cell’, but in some of the articles, SMC refers to ‘soil microbial community’ (e.g. PMID:28,620,371). False negatives are mainly due to the cell types which are not yet covered by CL such as cells tagged with cell surface markers, e.g., ‘CD58-positive natural killer cell’, and absent synonyms (e.g. ‘neuronal cell’ is not a synonym for ‘neuron’ in CL, although it is listed as synonym by alternative resources such as CellFinder [12]. Further false negatives are due to the limitations of our dictionary-based approach which fails to identify some entities that are characterized in natural language but not explicitly mentioned syntactically in the form used in our dictionary. For example, “hippocampal neurons” was identified while “cortical neurons” was not identified in a sentence containing “cortical and hippocampal neurons”. Overall, our cell type tagger achieved a precision value of 90.56%, a recall value of 60.00% and an F-score value of 72.18%. Results show that there is still some room for improving especially the coverage of CL which can be achieved by integrating additional synonyms for the existing cell types as well the new cell types available from other resources such as CellFinder.

### Cell type and cell line names usage in literature

Using our text mining approach, we were able to identify 1277 of 2180 (59%) cell types and 4907 of 38,605 (13%) cell lines in the open access full text articles.

**Fig. 1:**
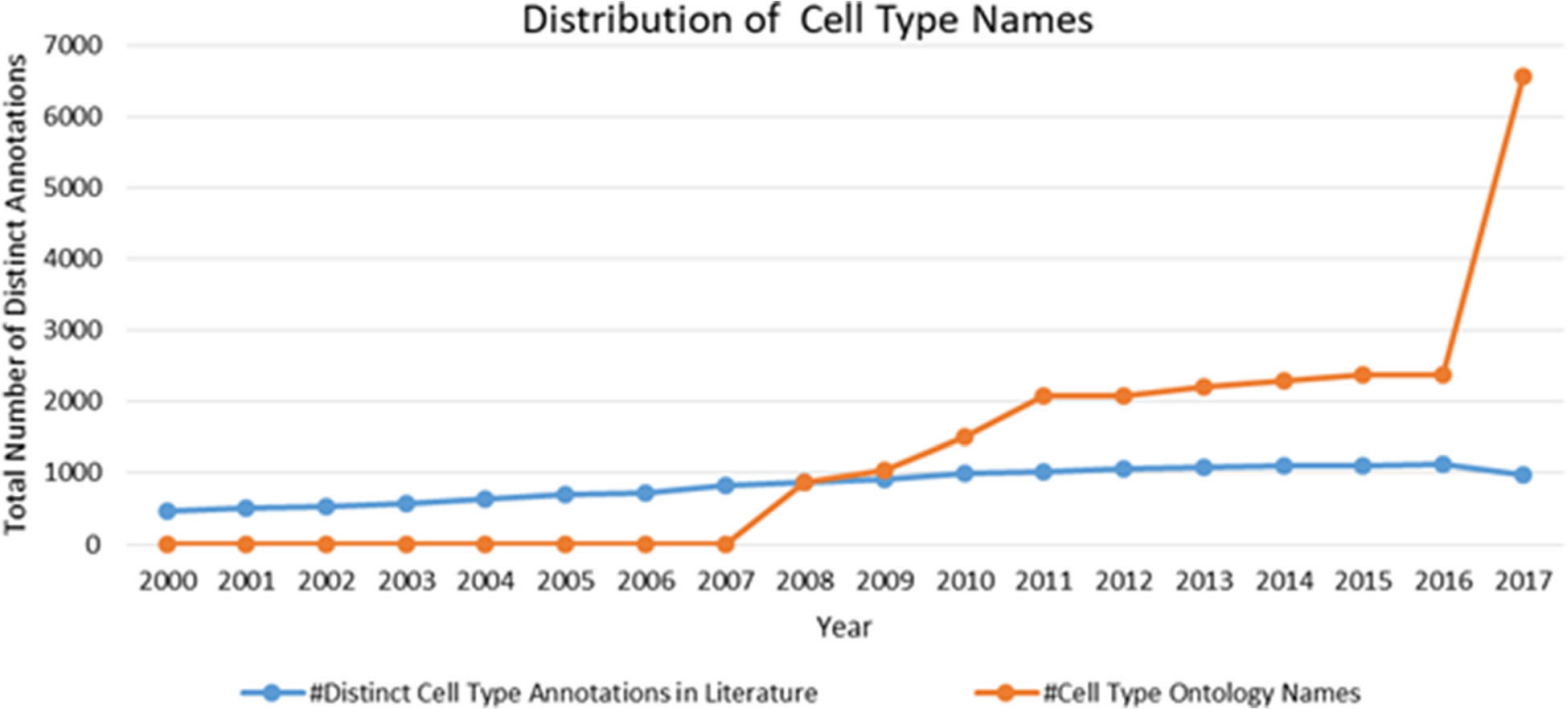
Distribution of cell types. 2017 data is as-of May 30th

**Fig. 2:**
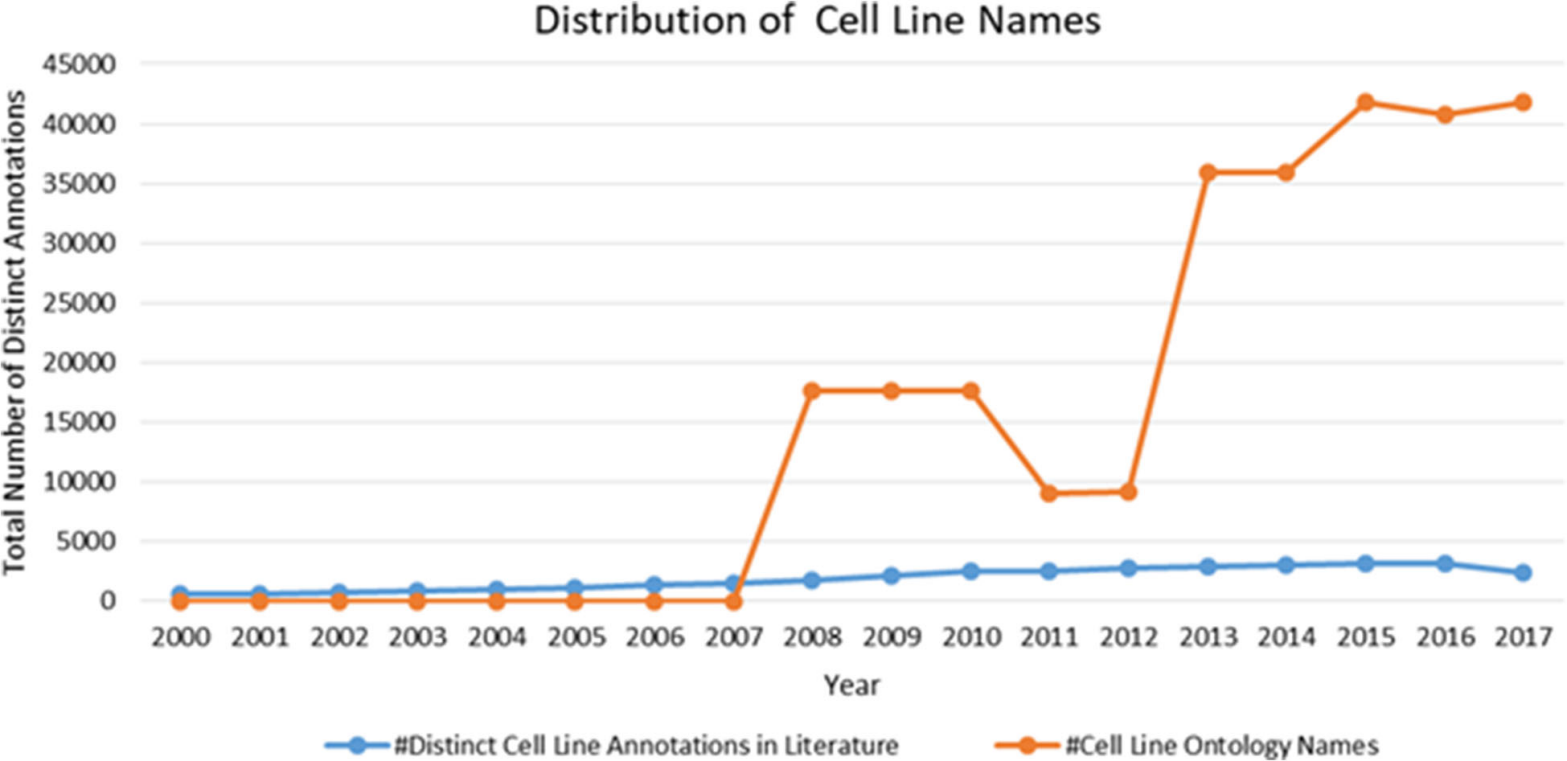
Distribution of cell types. 2017 data is as-of May 30th

Figures 1 and 2 show the distribution of the number of distinct cell type and cell line annotations between 2000 and 2017 in the open access full text articles respectively. As can be seen from the figures, the distinct number of annotations for both cell types and cell lines increases over the years showing an increasing uptake of the nomenclature by the scientists. Both, CL and CLO are released in 2008 and updates to the ontologies are released irregularly. As illustrated here, the development of these ontologies is slightly accelerated compared to the usage of cell nomenclature in literature; both now cover significantly more cell types and cell lines than are mentioned in text. In part, this may be due to the use of the ontologies for annotation of data resulting from high-throughput experiments, where the data is primarily found in databases and not explicitly mentioned in literature. In addition, we find that the usage of cell types in literature is more standardised compared to the usage of cell lines, as we could only find 13% of the cell lines from CLO using our text mining approach. Alternatively, CL could also cover more of the lexical variants and synonyms for cell types than CLO does for cell lines. Both cell line as well as cell type nomenclature suffers from a lack of authority in naming convention unlike other biological entities that have well-established consortium such as HUGO Gene Nomenclature Committee (HGNC) for gene names [13], or the Internal Union of Pure and Applied Chemistry (IUPAC) [14] for chemical names. Therefore, the use and generation of cell line names are often done in an ad-hoc manner without any enforcement of standardisation resulting in polymorphic spellings and formatting of the cell line names, which in turn impacts the recovery of synonymous terms.

Our analysis can also make artefacts resulting from the evolution of the ontologies visible. The drop in the number of CLO classes in 2011 is due to a restructuring of CLO resulting from the transformation of the original Cell Line Knowledgebase (CLKB) [15] cell line instances to classes in the CLO at a later time, classes that were removed in this process were re-added, resulting in higher coverage after completing the restructuring of the ontology.

**Fig. 3:**
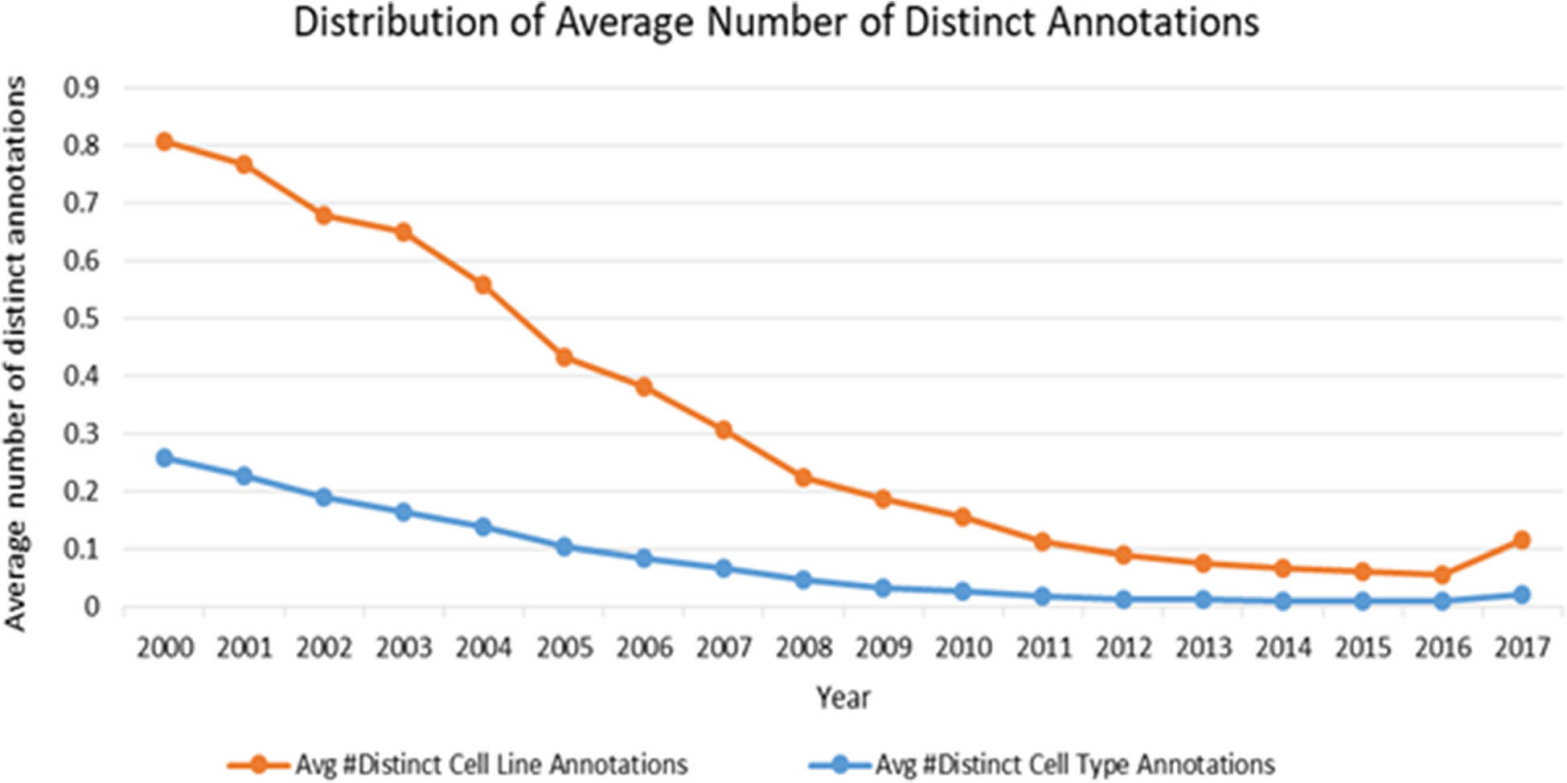
Distribution of average number of distinct annotations. 2017 data is as-of May 30th Average value per year is the ratio between the number of distinct annotations and the number of annotated articles

**Fig. 4:**
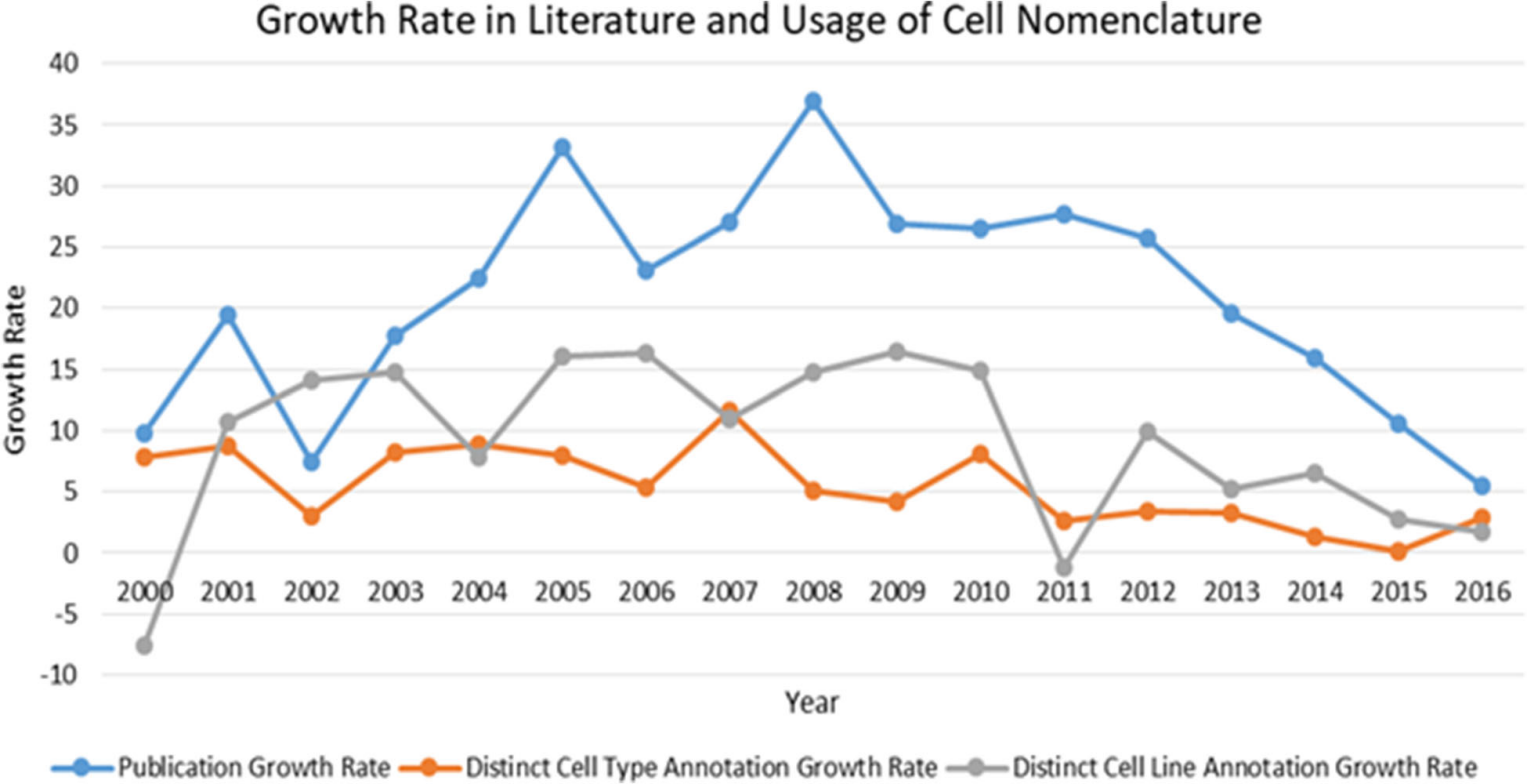
Growth rate (%) in literature and usage of cell nomenclature

As can be seen from Fig. 3, the average number of distinct annotations per article for both cell lines and cell types decreases over the years. The decreasing average values in Fig. 3, can be explained by analysing the growth rate in literature as well as in the cell nomenclature usage. As can be seen from Fig. 4, the growth rate (%) in literature is much steeper than the growth rate in the usage of both cell line and cell type nomenclature leading to decreasing average number of cell nomenclature annotations per article over the years. Reasons may include a broader coverage of different scientific disciplines within our corpus of open access articles as well as a move of the scientific community towards other types of experimental models beyond cell lines, in particular animal models [16].

### Representation of CL and CLO in literature

**Fig. 5:**
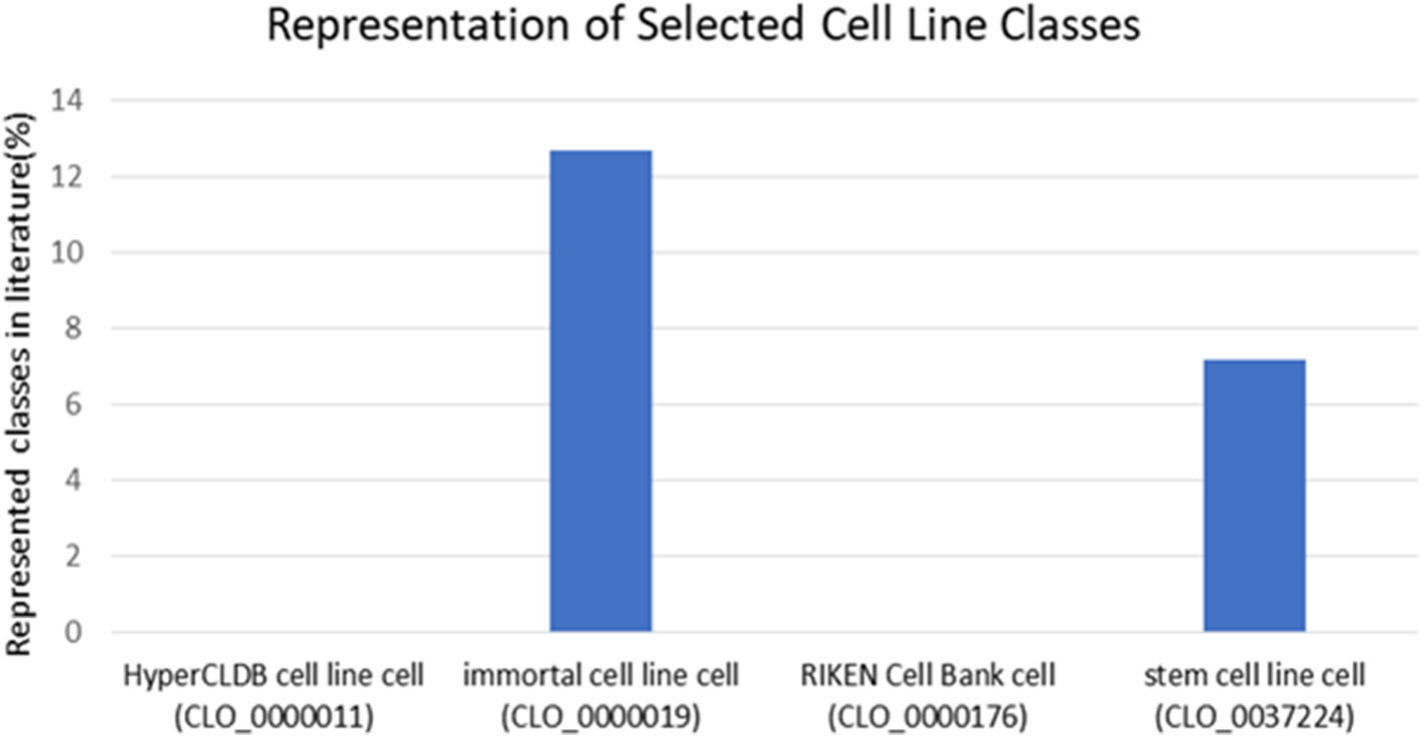
Representation of selected “cell line cell” classes in literature. Class representation of each class is calculated as the ratio between its number of subclasses referred to in the literature and its total number of subclasses

We projected the text mined annotations generated in this study (1277 cell types (59%) and 4907 cell lines (13%)) onto CL and CLO to reveal which branches of the ontologies are well covered and which branches are not referred to in the literature. This analysis is intended to highlight the ontology branches that need to be expanded, either by adding classes or their synonyms, as they are frequently referred to in literature but not available for ontology-based annotations. . Our analysis revealed that, for both CL and CLO, the majority of the classes are referred to in literature by their 10% or less only. Particularly, for CLO which is only 13% of it is referred to in the literature, almost all of the referred classes are represented by their 10% or less. This is potentially a strong signal for the existence of other cell line synonyms or classes preferably used by scientists and need to be added to the ontology. Several poorly represented ‘cell line cell’ (HyperCLDB cell line cell, immortal cell line cell, RIKEN cell Bank cell and stem cell line) classes are shown in Fig. 5, where we could identify 12% of their subclasses or less only. Some of the well represented CLO classes are ‘immortal human esophagus-derived cell line’ (CLO_0000656) and ‘immortal stomach-derived cell line’ (CLO_0000249), for which we identify mentions for their 34/37 (91.89%) and 40/45 (88.89%) subclasses, respectively cell).

**Fig. 6:**
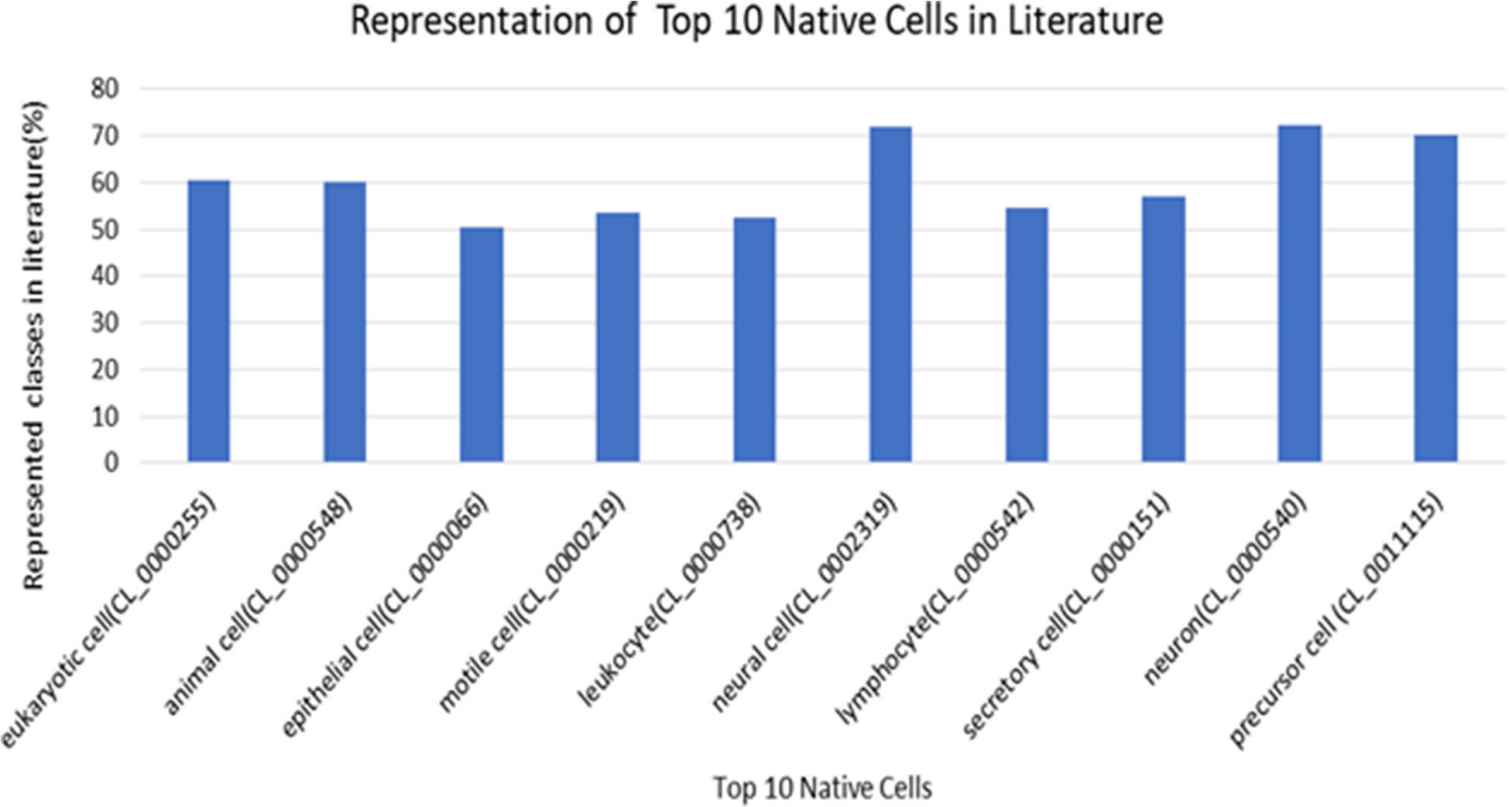
Representation of top 10 native cells in literature. Class representation of each class is calculated as the ratio between its number of subclasses referred to in the literature and its total number of subclasses

Although, throughout this study, we observed that the terminology underlying CL ontology is better standardised than the terminology underlying CLO, there are still many underrepresented classes which need to be analysed further for their expansion with possible synonyms. For example, as can be seen from Fig. 6, majority of the top 10 native cells (selected based on their number of subclasses) are represented by their 60% of the subclasses or less only in the literature. Some of the well represented CL classes are ‘glial cell (sensu Vertebrata)’ (CL_0000243) and ‘germ cell’ (CL_0000586) where 29/31 (93.55%) and 25/27 (92.59%) of their subclasses are found in the literature based on our text mining analysis.

A complete list of class representation of each individual class is available at https://github.com/shenay/CellNomenclatureStudy which can be used to prioritise the ontology classes that need to be focused for their further expansion with additional synonyms or sub classes.

### Outcomes and suggestions

The outcomes of our study can provide insights to improve and develop text mining methods so that cell lines and cell types can be captured in literature more accurately, and further lead to guidelines for continuous development of biomedical ontologies as well as recommendations for the use of cell type and cell line nomenclature in literature so that unambiguous understanding, which is a prerequisite for reproducibility, is ensured.

Our results will be of most interest to researchers in biomedical text mining*.* We generated a novel corpus annotated with mentions of cell types and cell lines, which can be used for developing and evaluating text mining methods. For example, our corpus can be used for training of named-entity recognition and normalisation systems that utilise machine learning approaches, as well as for evaluation of existing named entity recognition and normalisation approaches. Furthermore, these datasets can be expanded by using the dictionary-based taggers that we developed, an approach that would be justified based on the high precision our method achieves.

Our gold standard corpus may also serve to improve recall by utilizing the positive and negative annotations in the corpus, in a machine learning based annotation tool that learns to distinguish positive and negative occurrences of tokens that may refer to cell types or cell lines based on context. Such an approach would be particularly useful for cell lines as we found the cell line terminology to be highly ambiguous.

Our manual analysis further revealed that there are several cell type and cell line names missing in CL and CLO, respectively, which currently might be covered by other resources. Therefore, existing cell line and type resources should be merged to develop a comprehensive dictionary of names for cell biology, which can then be utilised to develop more comprehensive dictionary-based annotation tools.

The lack of an authority in cell line naming, or cell line naming conventions, leads to the frequent usage of ambiguous names. This brings limitations to efficient text mining application development.

For ontology developers, our most important finding is a set of missing cell type and cell line names and synonyms in CL and CLO. The ontologies can be improved by adding these synonyms and labels, for example by comparing the ontologies’ current content against other available cell type and cell line resources and adding the ones which are covered by the other resources but not by CL or CLO. Furthermore, our analysis shows that scientists sometimes create new names for entities used in their studies without explicitly reusing names already covered by standard resources. Using a machine learning based system to identify cell line and cell type names in text could reveal additional synonyms and new names that can be used for expanding the ontologies.

Further manual analyses either on the dictionary-based annotated or machine learning based annotated text would reveal preferred names by the scientist which should be used for refining the existing labels and synonyms in the ontologies. Additionally, our analysis on the distribution of the text mined cell line and cell type annotations based on the ontology classes uncovers the well or poorly represented classes in the literature. Outcomes of such this analysis can be used to refine the terminology used in the ontologies.

In the interest of reproducibility of research results, it would be beneficial if authority for naming convention for cell lines would be established. Alternatively, scientists should be encouraged to consider the usage of a given name in their publications if it already exists in standard resources such as the CLO.

For a new cell type or cell line which is not covered by standard resources, scientists should consider clear and effective communication while naming their entity. Currently, there is an overlap in names between cell types or cell lines and gene and protein names as well as with names used in other domains, which is a bottleneck in efficient scientific communication and information dissemination in the domain. Further analysis that can be conducted on our results could support new discoveries. For example, text mining can be applied on cell types and cell phenotypes to reveal their relations with diseases such as cancer.

Last but not least, scientists, ontology developers and text miners should follow the FAIR principles (Findability, Accessibility, Interoperability and Reusability) [17] to either produce and publish data or develop tools for maximising the added-value gained by research efforts. For this purpose, it is beneficial for each party to carry out transparent, reproducible and reusable research outcomes, and using unambiguous identifiers and terminology is a key component in achieving this goal.

## Conclusions

We performed a large-scale analysis of cell nomenclature usage in the biomedical literature. To this end, we used dictionary-based text mining methods to analyse the terms from CL and CLO in publications. Our approach identifies 59% of the cell type classes in CL and 13% of the cell line classes in CLO in the literature. We find that cell line nomenclature is much more ambiguous compared to the cell type nomenclature. Nevertheless, there is an increasing adoption of standardised nomenclature for cell lines by the scientists.

Our study presents insights into the past and current trends for the cell nomenclature usage in the biomedical literature and helps better standardisation of the nomenclature in knowledge bases as well as supports development of text mining applications relevant to cell types and cell lines.

In future, we plan to expand our analysis by covering other cell lines and cell types available in resources such as Cellosaurus and CellFinder to gain deeper insights into how CL and CLO might be improved.

## Abbreviations

CL: Cell Ontology
CLKB: Cell Line Knowledgebase
FAIR: Findability, Accessibility, Interoperability and Reusability
HCA: Human Cell Atlas
LO: Cell Line Ontology
MALLET: MAchine Learning for LanguagE Toolkit OBO – Open Biomedical Ontologies
